# The interactive role of type 2 diabetes mellitus and E-selectin S128R mutation on susceptibility to coronary heart disease

**DOI:** 10.1186/1471-2350-8-35

**Published:** 2007-06-20

**Authors:** Khaled K Abu-Amero, Futwan Al-Mohanna, Olayan M Al-Boudari, Gamal H Mohamed, Nduna Dzimiri

**Affiliations:** 1 Genetics Department, King Faisal Specialist Hospital and Research Centre, Riyadh 11211, Saudi Arabia; 2 Biological and Medical Research Department, King Faisal Specialist Hospital and Research Centre, Riyadh 11211, Saudi Arabia; 3 Biostatistics, Epidemiology and Scientific Computing Department, King Faisal Specialist Hospital and Research Centre, Riyadh 11211, Saudi Arabia

## Abstract

**Background:**

The role of gene-environment interactions as risk factors for coronary heart disease (CAD) remains largely undefined. Such interactions may involve gene mutations and disease conditions such as type 2 diabetes mellitus (DM2) predisposing individuals to acquiring the disease.

**Methods:**

In the present study, we assessed the possible interactive effect of DM2 and E-selectin S128R polymorphism with respect to its predisposing individuals to CAD, using as a study model a population of 1,112 patients and 427 angiographed controls of Saudi origin. E-selectin genotyping was accomplished by polymerase chain reaction (PCR) amplification followed by *Pst*I restriction enzyme digestion.

**Results:**

The results show that DM2 is an independent risk factor for CAD. In the absence of DM2, the presence of the R mutant allele alone is not significantly associated with CAD (p = 0.431, OR 1.28). In contrast, in the presence of DM2 and the S allele, the likelihood of an individual acquiring CAD is significant (odds ratio = 5.44; p = < 0.001). This effect of DM2 becomes remarkably greater in the presence of the mutant 128R allele, as can be observed from the odds ratio of their interaction term (odds ratio = 6.11; p = < 0.001).

**Conclusion:**

Our findings indicate therefore that the risk of acquiring CAD in patients with DM2 increases significantly in the presence of the 128R mutant allele of the E-selectin gene.

## Background

Coronary artery disease (CAD) is a complex disorder resulting partly from interactions between genetic risk factors and environmental components. Genetic risk factors include, among others, mutations in genes that play a role in disease manifestation, while environmental variables may involve events leading to functional changes in vascular reactivity or lipid metabolism, for example. E-selectin is a cell adhesion molecule that mediates neutrophil, monocyte and memory T-cell adhesion to cytokine-activated endothelial cells. It is expressed in endothelial cells at inflammation sites and plays a crucial role in monocyte trafficking [1]. Mononuclear cells isolated from insulin-resistant subjects have been reported to bind to endothelial cells with enhanced affinity [2-4]. Although the mechanisms are still poorly understood, this process seems to be modulated by various cell-adhesion molecules and might partly explain the increased risk of CAD associated with insulin resistance [3]. In particular, the S128R polymorphism (SNP rs5361) of the E-selectin gene has been associated with CAD, premature CAD, as well as type 2 diabetes mellitus (DM2). Thus, several studies involving various ethnic groups including Germans, Japanese, Americans, Chinese, Africans and Arabs have implicated the E-selectin 128R mutant allele as a risk factor for CAD [5-12]. Others have identified this allele as a possible marker for post-angioplasty restenosis in CAD patients [13].

In contrast, the relevance of E-selectin S128R polymorphism as a risk factor for diabetes is still not fully elucidated. One study involving first degree relatives has indicated that soluble E-selectin levels are elevated in patients with DM2 carrying the 128R allele in comparison with their non-diabetic relatives [10]. While a number of studies have also associated elevated soluble E-selectin levels with this mutation and identified it as a risk factor for DM2 in general [14-16], others seem to point to a gender (female)-related association of the soluble E-selectin levels with the disease [17-21], independent of obesity and elevated levels of inflammatory markers or other cell-adhesion molecules. In contrast to the above reports, a study by Meigs and colleagues suggested that E-selectin variants are not important genetic risk factors for DM2 in women [14]. Put together, it appears that the role of soluble E-selectin and/or its gene polymorphism in DM2 may depend on certain yet undefined confounding factors. Besides, it is still unclear whether the elevation in soluble E-selectin levels in such patients is directly linked to the S128R mutation or not.

Thus, while the role of DM2 is well-established as an independent risk factor for CAD, the available data on the influence of the E-selectin in CAD patients with DM2 is still partly controversial. Currently, the literature evaluating this possibility is limited and somewhat contradictory. For example, a study by Endler and others suggested that the 128R allele is not associated with CAD or an increased risk for myocardial infarction in patients with DM2 [22]. Even less is known about possible interaction between the E-selectin polymorphism and DM2 as potential predisposing factor for CAD. Nonetheless, the likelihood that both the S128R polymorphism and DM2 may individually constitute risk factors for this vascular disease implies that these two components may exert a synergist or additive effect of predisposing individuals to acquiring the disease. The objective of this study was, therefore, to assess the possible interactive effect of the E-selectin S128R polymorphism and DM2 as a risk for acquiring CAD in a relatively large group of patients from a homogenous Saudi population.

## Methods

### Study population

Two groups of Saudi individuals were recruited for the present study. The patient group comprised 1112 candidates (767 males and 345 females; mean age 54.2 ± 11.9 yr) of Saudi Arabian descent with angiographically documented CAD. The inclusion criterion for CAD was the presence of angiographically determined narrowing of the coronary vessels by at least 70%. Exclusion criteria for CAD were major cardiac rhythm disturbances, incapacitating or life-threatening illness, major psychiatric illness or substance abuse, history of cerebral vascular disease, neurological disorder, and administration of psychotropic medication. A second group of 427 individuals (238 males and 189 females, mean age 55.6 ± 11.8 yr) undergoing surgery for heart valvular diseases and those who reported with chest pain, but were established to have no significant coronary stenosis by angiography, were recruited as angiographed controls (CON). Exclusion criteria for this group included among others diseases such as cancer, autoimmune disease, or any other disorders likely to interact with variables under investigation. Diabetic patients either had a known history of type 2 diabetes mellitus or were diagnosed according to the American Diabetes Association criteria [23]. Full informed consent was obtained from all patients or family members before participating in the study. This study was performed in accordance with the Declaration of Helsinki as adopted and promulgamated by the US National Institutes of Health, as well as rules and regulations laid down by the Hospital's Ethics Committee.

### Detection of the E-selectin S128R polymorphism

Five ml of peripheral blood were collected in EDTA tubes from all participating individuals. DNA was extracted using the Puregene kit from Gentra Systems (Minneapolis, MN, USA), and stored in aliquots at -20°C until required.

The determination of E-selectin polymorphism was carried out by polymerase chain reaction (PCR) amplification followed by *Pst*I restriction enzyme digestion, as described previously [8,24]. The sizes of the digested amplicons were determined using the 50-bp ladder (Amersham Pharmacia Biotech, Piscataway, NJ, USA). The A > C nucleotide change at position 561 of the E-selectin gene (corresponding to S > R amino acid change at codon 128) abolishes a recognition site for the *Pst*I restriction enzyme. As a result, homozygous A/A (S/S) genotype produces two fragments of 123 and 63 bp in size following restrictive digestion with this enzyme. Heterozygous A/C (S/R) genotype results in three fragments of 186, 123 and 63 bp, and homozygous C/C (R/R) genotype results in one fragment of 186 bp. As a quality control, we confirmed by direct sequencing the genotype status of 384 random samples representing the three different genotypes.

### Statistical analysis

Analysis for the prediction of CAD was performed using logistic regression. Gene-environment interaction terms were estimated according to Yang and Khoury (1997) [25]. All statistical analyses were performed using the SPSS software version 14 (SPSS Inc., Chicago, USA). A two-tailed *p *value < 0.05 was considered statistically significant.

## Results

In assessing the interaction of E-selectin genotypes with diabetes (environment), we followed the approach of Yang and Khoury (1997). In this model the genotype is dichotomous (carriers versus non-carriers of E-selectin) and the environmental exposure (diabetes) is dichotomous (diabetic versus non- diabetic). In the present case, the four possible combinations of genotype and exposure can be displayed in a 2 × 4 table, and three categories of joint exposure can be compared with a reference category (for which the relative risk is by definition, 1.0). The relative risk of developing CAD in individuals who are both genetically susceptible to the condition and have been exposed to the environmental variable compared to the reference group will be the measure of interaction. Thus, the variable assumes the value of 1 (reference group) in the absence of both diabetes and the variant S allele, the value of 2 in the presence of both diabetes and the S allele, 3 in the absence of diabetes and presence of the mutant R allele, and 4 in the presence of both diabetes and the R allele.

The clinical and demographic data of the patients and control subjects are given in Table 1. As indicated in this table, the cases and control groups are generally comparable with one another. As describe above, Table 2 gives the interaction of DM2 and E-selectin polymorphism as a combined risk factor for CAD. The table demonstrates that in the absence of DM2, the presence of R mutant allele does not have a significant effect on the development of CAD (p = 0.431, OR 1.28). In the presence of DM2 and the S allele, the likelihood of acquiring CAD is significant (odds ratio = 5.44; p = < 0.001). Furthermore, in the presence of the R mutant allele and DM2, the odds ratio increases from 5.44 (in presence of the wild type S allele) to 6.11 (p = < 0.001). Since DM2 is an independent risk factor for CAD (p < 0.0001, Table 1), these results point to an augmentation of the effect of DM2 by the presence of the R allele on individual's susceptibility to CAD.

**Table 1:** Clinical and demographic characteristics of the control and CAD groups

		**Controls**	**CAD**
Total		854	1112
Mean age (± SD)		55.7 ± 11.8	54.2 ± 11.9
Male		238 (55.7)	767 (69.0)
Smoking		191 (44.7)	344 (30.9)
Diabetes		287 (67.2)	1019 (91.6)
Hypertension		268 (62.8)	929 (83.7)
Family history		95 (22.4)	437 (39.7)
High cholesterol		138 (32.5)	895 (80.7)
High triglycerides		67 (15.7)	780 (70.1)

The table shows the important clinical features of the studied groups. The numbers in brackets give the percentages of the given values. P-value chi by square test is 0.0001 for all variables except age, which gave p-value of 0.032 using Student's t-test.

**Table 2:** Interaction of diabetes mellitus and E-selectin polymorphism in coronary artery disease

**Interaction**	**Total**	**Controls n = 854**	**CAD n = 2224**	**odds ratio (95% C.I)**	**P value**
No diabetes * S allele	422	256	166	Reference	-
No diabetes * R allele	44	24	20	1.28 [.69–2.4]	0.431
diabetes * S allele	2458	543	1915	5.44 [4.4–6.7]	< 0.001
diabetes * R allele	154	31	123	6.11 [3.9–9.5]	< 0.001

The data for the interaction between the S128R mutation and type 2 diabetes mellitus with regard to susceptibility of diabetic patients to acquiring coronary artery disease are presented in a 2 × 4 table. The four possible combinations of genotype and exposure are given in the first column of the table, and three categories of joint exposure are compared with the reference category of No diabetes * S allele.

We further performed multiple logistic regression analysis to adjust for potentially confounding variables. Thus, in addition to the variable representing the interaction between diabetes and E-selectin, we included cholesterol, triglyceride, gender, age, hypertension, family history of CAD and smoking (Table 3). The odds ratio for the interaction between diabetes and E-selectin polymorphism adjusted for all these variables was 6.41 (95% CI 3.61 – 11.37), which is similar to the results of the univariate analysis given in Table 2.

**Table 3:** Multiple logistic regression analysis for the interaction between E-selectin genotypes and other risk factors for coronary artery disease

**Variables**	**Odds Ratio**	**95% C.I. for odds ratio**	**p-value**
Gene – Diabetes interaction			
no diabetes S (reference)	1	-	-
no diabetes R	1.11	0.49–2.53	0.79
Diabetes S	6.06	4.17–8.78	0.0001
Diabetes R	6.41	3.61–11.37	0.0001
Cholesterol	2.01	1.57–2.55	0.0001
Triglycerides	6.49	4.98–8.46	0.0001
Gender	1.57	1.27–1.93	0.0001
Age	0.98	0.97–0.99	0.0001
Hypertension	2.08	1.66–2.59	0.0001
Family history	2.19	1.75–2.74	0.0001
Smoking	1.12	0.85–1.49	0.41

The table shows that the results of the univariate analysis in Table 2 are not affected by other confounding risk factors for coronary artery disease.

## Discussion

The present study investigated possible interaction between DM2 and the E-selectin S128R polymorphism in predisposing individuals to acquiring CAD, using a large homogenous Saudi population as a study model. The results indicate that the presence of DM2 alone constitutes a risk for CAD, as shown by the association between the two disorders in the presence of the wildtype S genotype. They also demonstrate that, while the mutant 128R alone is not associated with CAD, its presence significantly contributes to the potency of DM2 as a risk factor for acquiring the disease. The fact that adjustment for other risk factors for CAD did not alter the level of significance for the interaction observed in this study adds weight to the notion of its predictive power as being independent of confounding variables. Put together, therefore, it can be inferred that the presence of the mutant R allele of the E-selectin gene greatly increases the likelihood of patients with DM2 acquiring CAD.

While the importance of DM2 as an independent risk factor for CAD is well-established, the role of the E-selectin polymorphism is still somewhat controversial, despite several studies addressing its potential relevance as a risk factor for CAD and DM2, respectively. Thus, currently available data on the association of the 128R genotype with CAD is partly inconsistent, with some studies implicating it in disease manifestation [8,12,26-28], possibly on ethnic or gender basis, and others failing to establish such a relationship [22]. Even less convincing is the data on its role in DM2, where the general consensus appears to be equally divided between findings purporting an association [18] and those advocating the opposite [22].

It is noteworthy that both CAD and DM2 are independently thought to be characterized by endothelial dysfunction [19,29,30], and an elevation in soluble E-selectin levels has been identified as a biomarker for both disorders. This scenario points to a possible link between changes in the E-selectin levels, endothelial dysfunction and manifestation of both diseases. Furthermore, if the mutant 128R allele is involved in both CAD and DM2, it follows that a combined effect of this mutation and DM2 would pose at least an additive risk for acquiring CAD. To our knowledge, there is hardly any data in the literature pointing to an interaction of these variables in predisposing individuals to CAD. If anything, some current opinion seems to suggest that the 128R allele may not be associated with CAD or an increased risk for myocardial infarction in patients with DM2 [22]. This is somewhat at variance with our present findings, in that we have established an indirect association between the allele and CAD. Thus, particularly notable is our observation that the presence of the mutant genotype alone is not associated with CAD, entailing that its significance as a risk for CAD becomes apparent only in the presence of DM2. This scenario would have great implications for the possible mechanism of this interaction on the manifestation of CAD. To begin with, as mentioned above, an elevation in E-selectin levels appears to be a feature of certain groups of patients with CAD, restenosis as well as DM2. The question remains whether or not this elevation is due to the S128R mutation. While a large number of studies available in the literature have associated changes in DM2 or CAD primarily with either an increase in E-selectin levels or S128R polymorphism, only a few of these investigations have addressed this issue directly. The discrepancies in these association studies also imply that changes trigerred by the mutation may be partly discernible from those resulting from elevated E-selectin levels. In this case, the influence of the mutation may underlie various mechanisms in different disorders. Interesting in this regard is a recent study by Jilma and colleagues in a human model of endotoxin-induced tissue-factor-triggered coagulation which found that the S128R mutation has no significant influence on the basal or inducible soluble E-selectin, but enhances thrombin generation substantially [31]. These authors concluded that this coagulant effect may contribute to the linkage of this polymorphism with various thrombotic cardiovascular disorders [31,32]. A potential scenario, therefore, is the likelihood that the function of the mutation in the cardiovascular disorders, such as CAD, might be related to a prothrombotic action mechanism, which may become particularly prominent in the presence of DM2. Hence, although some studies seem to suggest an ethnic or a gender component of the role of the S128R polymorphism on these diseases, it would appear that the impact of this mutation is essentially linked to the prevalence of DM2, rather than being peculiar to certain ethnic groups.

However, it should be noted that the genetic architecture of atherosclerosis and/or diabetic macrovascular complications is likely to result from the contribution of many genes interacting with different environmental factors. An undoubted limitation of our study is the lack of more comprehensive genetic analysis of potential candidate genes. The availability of recently developed assays capable of simultaneously genotyping multiple loci should offer appropriate approaches for the screening of genotype combinations of candidate genes to identify diabetic patients at a high risk of macro-complications.

## Conclusion

In conclusion, the present study points to a possible interaction of the E-selectin S128R polymorphism and type 2 diabetes mellitus in predisposing individuals to acquiring coronary heart disease. While further investigations are warranted to confirm our findings, we nonetheless believe that our findings will contribute to the understanding of the molecular mechanism underlying the association of this mutation with DM2 and CAD.

## Competing interests

The author(s) declare that they have no competing interests.

## Pre-publication history

The pre-publication history for this paper can be accessed here:


